# Elucidating the Correlation of D-Dimer Levels with COVID-19 Severity: A Scoping Review

**DOI:** 10.1155/2022/9104209

**Published:** 2022-03-08

**Authors:** Wesam Ahmed Nasif, Abeer Shaker El-Moursy Ali, Mohammed Hasan Mukhtar, Aali Marzouq H. Alhuzali, Yahya Ahmed Yahya Alnashri, Ziyad Ishaq Ahmed Gadah, Eyyad Adeeb A. Edrees, Hussam Abdulaziz Mabruk Albarakati, Hussam Saud Muhji Aloufi

**Affiliations:** ^1^Biochemistry Department, Faculty of Medicine, Umm Al-Qura University, Mecca, Saudi Arabia; ^2^Molecular Biology Department, Genetic Engineering and Biotechnology Research Institute, Sadat City University, Sadat City, Egypt; ^3^Department of Pathology, Faculty of Medicine, Umm Al-Qura University, Al-Abdia Main Campus, Mecca, Saudi Arabia; ^4^Faculty of Medicine, Umm Al Qura University, Makkah, Saudi Arabia

## Abstract

**Aims:**

The review explores the findings of previous studies to elucidate the association between levels of D-dimer and COVID-19 severity and prognosis. In addition, we assessed the efficiency of anticoagulant therapies in reducing COVID-19 severity and improving the prognosis of the patients.

**Materials and Methods:**

A comprehensive literature review was conducted using MEDLINE/PubMed databases, Scopus, and Web of Science with the help of keywords “COVID-19,” “D-Dimer,” “Thrombosis,” “Fibrin network,” “Anticoagulant therapy,” “Inflammation,” and “disease severity.” Based on all these articles and clinical experience, a scoping review was constructed and the full texts of the articles that were retrieved were accessed.

**Results:**

A D-dimer is a complex protein molecule that is formed during plasmin-mediated degradation of the fibrin network. Thus, it serves as a marker of thrombotic activity. On the other hand, in addition to severe respiratory distress and reduction in pulmonary gas exchange, severe acute respiratory syndrome coronavirus 2 (SARS-CoV-2) also triggers prothrombotic changes in the infected individuals. The levels of D-dimer have been postulated to be positively associated with the degree of disease severity among COVID-19 patients.

**Conclusions:**

It has been postulated that D-dimer could potentially be used as a biomarker to predict the prognosis and outcome of COVID-19 patients at the time of admission to hospitals and facilitate more personalized and efficient clinical management that could significantly reduce the mortality rate of such patients and allow more rapid recovery.

## 1. Introduction

The first case of coronavirus disease 2019 (COVID-19) was reported in December 2019 in the Wuhan province of China. After that, it quickly spread across 200 countries within a span of a few months. Global research efforts quickly identified the etiological agent of this disease to be a novel coronavirus. This novel coronavirus exhibited approximately 80% homology to SARS-CoV, which had earlier spread during 2002–2003 and was associated with acute respiratory distress syndrome (ARDS) and a high mortality rate [1]. The novel coronavirus was hence named severe acute respiratory syndrome coronavirus-2 (SARS-CoV-2 or 2019-nCoV). Although this virus entered the human population via zoonotic transmission, its spread is mainly attributed to human-to-human transmission [2]. The World Health Organization (WHO) declared this outbreak a Public Health Emergency of International Concern by 30^th^ January 2020. Later, this outbreak was declared a global pandemic by 12^th^ March 2020 [3].

The novel coronavirus primarily transmits via respiratory droplets. The angiotensin-converting enzyme 2 receptor present on the cell surfaces plays a key role in the invasion of the novel coronavirus [4]. Most of the COVID-19 patients are usually asymptomatic and do not require hospitalization. However, sometimes, after an incubation of 2–14 days, COVID-19 symptoms may emerge, including shortness of breath, fever, coughing, and pneumonia [5]. Previous studies have reported that some cases exhibit the development of severe pneumonia, leading to hypoxia and respiratory dysfunction. The most severe cases (less than 5% of the symptomatic cases) exhibit ARDS and multiple organ failure, along with several laboratory abnormalities, such as leukopenia, thrombocytopenia, hypercoagulative state, leukopenia, and elevated levels of D-dimer, which often warrant admission to the intensive care unit (ICU) [6, 7]. This review focuses on the potential of D-dimer as a biomarker to predict the disease severity of COVID-19 patients and their outcome. We also assessed the efficiency of anticoagulant therapies among such patients to reduce their disease severity and improve their prognosis.

## 2. Materials and Methods

A comprehensive literature review was conducted using MEDLINE/PubMed databases, Scopus, and Web of Science with the scope of the searches confined to English language sources. There was no time restriction specified since the items on COVID-19 continue to accumulate on a regular basis. Priority was given to articles with a stronger evidence base, even though all types of articles were examined. The following keywords were employed: “COVID-19,” “D-Dimer,” “Thrombosis,” “Fibrin network,” “Anticoagulant therapy,” “Inflammation,” and “disease severity.” In general, we followed the guidelines for producing scoping reviews that were provided to us.

## 3. Result

### 3.1. D-Dimer Structure and Formation

**Figure 1:**
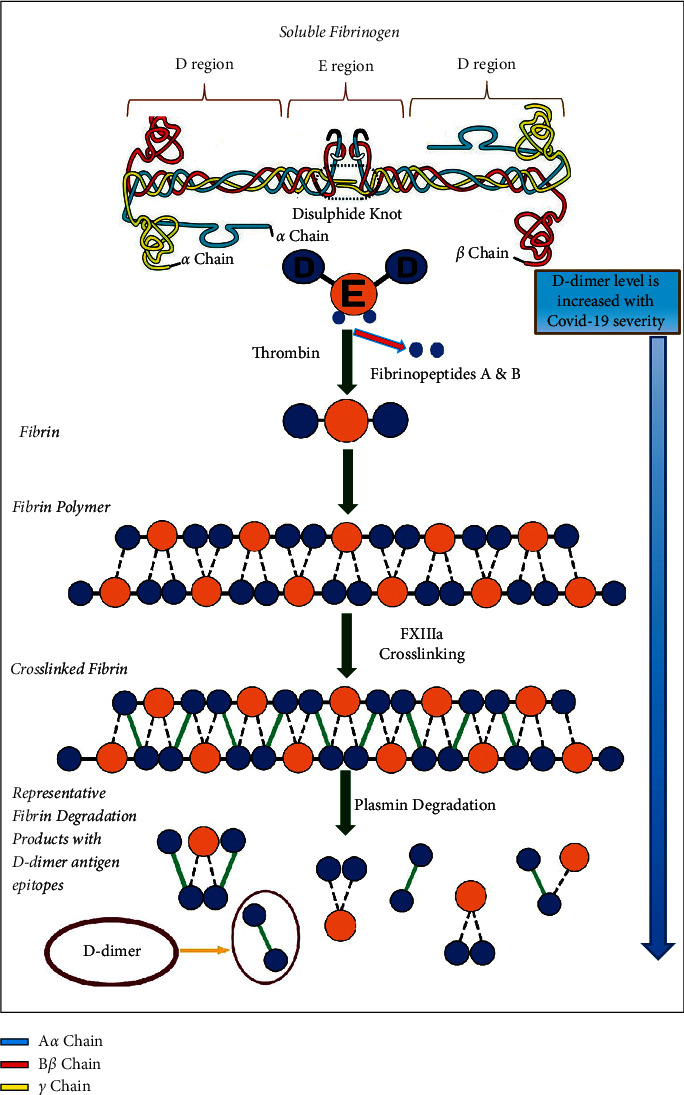
Schematic illustration of Fibrin structures of human (A) fibrinogen and (B) fibrin fibers. The fibrin structure was constructed by performing a best fit of each end of fibrinogen to each D region of the D dimer. Fibrinogen is clotted by thrombin, and the fibrin monomers that are produced polymerize spontaneously in a half-staggered format into protofibrils. The fibrin network is enhanced by factor XIIIa, which crosslinks adjacent monomers. Plasminogen activation is enhanced with fibrin formation, and the resultant plasmin digests the individual fibers. Plasmin cleavage between the D and E domains yields (DD)E, and the noncovalent complex of D-dimer (DD) and fragment E. D-dimer level is increased with COVID-19 severity.

A D-dimer is a complex protein molecule that is generated during plasmin-mediated cross-linked fibrin degradation.  Figure 1 depicts the process of D-dimer formation in the form of a schematic illustration. As the D-dimer formation commences, the fibrin molecules are formed after the thrombin-mediated cleavage of fibrinogen, a soluble glycoprotein that is found in the plasma. Thrombin cleaves the polymerization site of a fibrinogen molecule, thereby exposing the site to bind with other fibrinogen or fibrin molecules. In this manner, several such cleaved fibrin molecules bind together in an overlapping fashion to form protofibrils [8]. The thrombin molecules remain bound to the fibrin molecules during their polymerization. At this point, thrombin simultaneously activates the fibrinogen-bound plasma factor XIII. The complex of the thrombin molecules, plasma factor XIII, and fibrin polymers together triggers the formation of factor XIIIa [9]. Plasma factor XIIIa is instrumental in the cross-linking of the fibrin molecules. In the next step, plasminogen interacts with the fibrin molecules, which leads to the formation of plasmin.

The generated plasmin molecules, in turn, bind with the fibrin molecules and mediate degradation of the bound fibrin into products with different molecular weights, commonly known as fibrinogen degradation products (FDPs). Plasmin also mediates terminal degradation of the cross-linked fibrin molecules into soluble fragments that contain DDE fragments. A DDE fragment, simply put, is a D-dimer molecule that is noncovalently bound to fragment E. The plasmin molecule further breaks down the DDE fragment into DD and E fragments. The D-dimer (DD fragment + E fragment) is a soluble complex and circulates in the plasma until it is eliminated by the reticuloendothelial and renal pathways [9]. It is noteworthy that the half-life of D-dimer that circulates in the plasma is 8 h and can be detected in the blood only 2 h after the formation of a thrombus [10]. The formation of D-dimers only occurs during the generation and degradation of the cross-linked fibrin molecules, which takes place during coagulation and fibrinolytic events. Therefore, the D-dimer molecules serve as a direct marker of these events as well as an indirect marker of thrombotic activity.

There are three major steps of D-dimer formation:

- Fibrin monomers are generated after the degradation of a fibrinogen molecule by thrombin. The generated fibrin monomers then bind to other fibrin or fibrinogen molecules, which results in the formation of protofibrils. The dotted lines between the D-E domain and the D domain depict the noncovalent interactions that aid in maintaining the structural integrity of the protofibrils.
- Simultaneously, thrombin activates the formation of plasma factor XIIIa that binds to the D-domains of the fibrin polymers via covalent interactions.
- Then, fibrin degradation products (FDPs) are formed after the disrupting action of plasmin on multiple sites of fibrin, which, in turn, exposes the D-dimer antigen epitope. These FDPs are then further degraded, resulting in the formation of a terminal DDE complex [9].

### 3.2. Cross-Link between Thrombosis and Fibrinolytic Pathways

The fibrinolytic system is responsible for the prevention of the formation of fibrin thrombi. Such thrombi are mainly composed of fibrin polymers and, under normal circumstances, they are disrupted by the fibrinolytic system as soon as they are generated. Currently, such FDPs are the most widely used biomarkers of thrombosis. Several modulators play a key role in the promotion (such as tissue plasminogen activator and TPA) or prevention (such as thrombin activatable fibrinolysis inhibitor) of fibrin degradation. As abovementioned, the DDE fragment (D-dimer) only generates when plasma factor XIII degrades the fibrin polymers. Thus, this fragment holds great potential as a biomarker of fibrin degradation and coagulatory pathways and, in turn, thrombosis [11].

### 3.3. COVID-19 and Thrombosis

Since its emergence, several investigators around the globe have published a plethora of studies on the epidemiology of COVID-19. Although its transmittance rate is extremely high, it has a very low mortality rate of 2.3% [12]. A higher incidence of COVID-19 has been reported among individuals aged more than 65 years and less than 18 years, with a higher sequential organ failure assessment (SOFA) score, male gender, and several comorbidities, including diabetes, hypertension, and coronary heart disease [3]. In addition, several studies have also shown that a D-dimer level of more than 1 *μ*g/mL to be a potential risk factor of COVID-19 [13]. The formation of thrombi has been reported in both the venules as well as the arterioles of COVID-19 patients at the time of hospitalization. It has been postulated that this observation could be attributed to the risk factors that contributed to the aggravation of COVID-19 in an individual, such as obesity, pregnancy, and comorbidities like diabetes mellitus, which often themselves participate in and trigger the formation of clots in the bloodstream [14, 15]. The formation of such thrombi and the resultant angiogenesis often give rise to impaired microcirculation in COVID-19 patients [16]. Previous studies on COVID-19 patients have shown that the novel coronavirus is often responsible for endothelial injury and cell membrane destruction. This, in turn, reduces the fibrinolytic activity of endothelial cells, which promotes the formation of thrombi [17].

It is noteworthy that the proinflammatory cytokines are involved in both inflammatory and coagulatory processes. Previous studies have shown that severe infection of novel coronavirus triggers severe inflammatory reactions, as indicated by the significant upregulation of proinflammatory cytokines [18]. The cytokine upregulation and coagulopathy observed in cases of severe novel coronavirus infection have been attributed to acute sepsis [19]. Furthermore, severe novel coronavirus infection often predisposes infected individuals to sepsis-induced coagulopathy and disseminated intravascular coagulation [20]. Recently, Maier et al. reported a significant association between novel coronavirus infection and an increase in the viscosity of the plasma of the infected individual [21]. Such an increase in plasma viscosity has been reported to be associated with an increase in SOFA scores. This finding indicated that hyperviscosity of the plasma could trigger both endothelial dysfunction as well as thrombosis. Blood viscosity is also affected by fibrinogen levels [22]. Previously, it has been reported that the fluidic state of the plasma is maintained only till thrombin is able to cleave about 25% to 30% of the plasma fibrinogen. This, in turn, facilitates efficient polymerization of fibrin monomers and promotes the activation of plasma factor XIII by thrombin [9]. It is demonstrated that COVID-19 patients also exhibited high plasma levels of fibrinogen [22].

## 4. Discussion

### 4.1. Association between Levels of D-Dimer and COVID-19

As stated above, the levels of D-dimer directly correlate with the rate of formation and degradation of plasmin. Hence, any pathological condition that upregulates the rate of plasmin generation and degradation would also increase the levels of D-dimer. Thus, the pathologies that promote chronic inflammation, such as rheumatoid arthritis, asthma, and cancer, also lead to an increase in the levels of D-dimer. It follows that infection of a novel coronavirus, which leads to upregulated inflammatory reactions among individuals, would also increase the levels of D-dimer. This is evident by the findings of several previous studies that showed that levels of D-dimer were significantly higher in COVID-19 patients, especially those who were either severely ill or had deceased [23, 24]. Some investigators have postulated that the upregulated levels of D-dimer in individuals with severe novel coronavirus infection might be associated with severe illness, higher rates of thrombotic activity, and higher mortality rates of such patients [14, 25].

In 2020, Guan et al. presented the results of a large retrospective study that indicated the correlation between abnormal levels of D-dimer and disease severity of the COVID-19 patients for the first time [26]. Setting a cut-off point of a D-dimer level of more than 0.5 mg/L, they reported that a significantly higher proportion of novel coronavirus infected individuals with severe illness exhibited abnormally high levels of D-dimer than those with only mild or moderate illness (*p*=0.002) [26]. Furthermore, Tang et al. reported that COVID-19 patients with a severe level of illness exhibited approximately 3.5 times higher levels of D-dimer compared to patients with only mild or moderate levels of illness [27]. Their results were corroborated by Wang et al., who reported approximately 2.5- and 5-times higher levels of D-dimer, respectively, in COVID-19 patients with a severe level of illness compared to patients with only mild or moderate levels of illness [23, 24]. In line with these findings, Wang et al. also found that the levels of D-dimer in COVID-19 patients with severe illness were more than two times lower compared to the levels of D-dimer of deceased COVID-19 patients. Their results were supported by Tang et al., who demonstrated that COVID-19 patients with severe illness exhibited around four- and nine-times lower levels of D-dimer, respectively, compared to the levels of D-dimer of deceased COVID-19 patients [13, 27].

In a recent study, Lippi and Favaloro observed that the levels of D-dimer of the COVID-19 patients with a mild or moderate level of illness, that is, those who did not require ICU admission, were significantly lower than the levels of D-dimer of the patients with a severe level of illness, that is, those who required ICU admission [28]. In another study, Yao et al. demonstrated that COVID-19 patients who were categorized as suffering from a severe level of illness on the basis of their oxygenation index, lung CT scans, and corresponding clinical guidelines exhibited a significant association between their disease severity and levels of D-dimer [18]. Bilaloglu et al. conducted a multicentric study to examine the upregulation of levels of D-dimer in individuals who were hospitalized due to COVID-19 [29]. Their results indicated that, among the recruited patients, around 76% of the patients exhibited abnormally high levels of D-dimer during admission to the hospital, whereas around 86% of the patients exhibited abnormally high levels of D-dimer at any time during their hospitalization. Petrilli et al. reported that, at the time of admission to the hospital, the COVID-19 patients with abnormal levels of D-dimer exhibited poorer outcomes [30]. Around 43%, 20%, and 45% of the patients suffered from acute kidney injury, thrombosis, and critical illness, respectively. In addition, they conducted a multivariate analysis to identify the underlying factors that affected the outcomes of such patients and found that the levels of D-dimer were independently correlated with the patient's outcome. On the contrary, patients with normal levels of D-dimer exhibited higher odds of recovering without developing severe illness.

The novel coronavirus infection leads to upregulation of the inflammatory pathways. According to the results of several previous studies, the abnormal increment in the levels of D-dimer under inflammatory conditions indicates that the upregulation of inflammatory reactions and proinflammatory agents might be associated with the induction of the coagulatory pathways [31]. Hence, it follows that the coagulatory events in the COVID-19 patients might be triggered by the upregulated inflammatory reactions. This postulate was further strengthened by the findings of Bilian et al., who reported a significant correlation of the levels of D-dimer with the levels of hsCRP, a marker of inflammation, in COVID-19 patients [32]. In another study, Chen et al. showed how the levels of D-dimer could potentially be used as a marker to predict the inhospital mortality rate of COVID-19 patients [33]. Based on their results, they were able to determine a cut-off D-dimer value of more than 2.14 mg/L to predict the outcome of COVID-19 patients at the time of admission to the hospital. They reported that this cut-off value could be efficiently used to predict the rate of inhospital mortality with 88.2% sensitivity and 71.3% specificity. On the other hand, in their recent review, Zheng et al. reported that the levels of D-dimer of more than 0.5 mg/L indicated abnormally high blood coagulability of COVID-19 patients and were significantly correlated with poor outcomes of such patients [34].  Table 1 enlists some of the retrospective studies that demonstrated a significant correlation between levels of D-dimer and disease severity among COVID-19 patients [13, 27, 35–43].

### 4.2. Future Implications: Scope of Anticoagulation Therapy

Considering the COVID-19 pandemic, identifying therapies that improve outcomes is crucial. The findings presented in this review indicate that D-dimer could prove to be a potential biomarker to assess the disease severity level of COVID-19 patients and predict their outcome. This postulate is further supported by the analysis of Sakka et al., who suggested that levels of D-dimer could be helpful in categorizing the COVID-19 patients in terms of their disease severity levels at the time of admission to the hospital itself. They further reported that such categorization of the patients could facilitate personalized and more efficient clinical management in a timely manner based on their disease severity level [44]. Furthermore, several studies have shown that novel coronavirus infections lead to a significant increase in the formation of thrombi in the vascular system of COVID-19 patients. These findings indicate that apart from being a marker of elevated coagulation activity and prothrombosis in COVID-19 patients, D-dimer could also be involved in the pathogenesis of the disease [7]. Owing to the significant association of levels of D-dimer with prognosis and outcome of COVID-19 patients, the latest guidelines of the International Federation of Clinical Chemistry and Laboratory Medicine suggest that physicians must consider examination of levels of D-dimer in individuals suffering from COVID-19 [18, 45]. Recently, Long et al. also warranted the need to modify the thromboprophylaxis protocol for COVID-19 patients who are hospitalized [46]. In another study, Barnes et al. suggested that the modified prophylaxis protocol of COVID-19 patients must include anticoagulant therapies commencing immediately after admission to the hospital to reduce the rate of thrombi formation in such patients, provided such therapies do not lead to the risk of bleeding among the patients [47]. In this direction, Spyropoulos et al. further suggested that the application of any such modified prophylaxis protocol should be followed by an examination of a modified venous thromboembolism risk score to assess whether the patients are benefitting from the modified protocol [48].

Currently, the COVID-19 patients are managed using pharmacological DVT prophylaxis. However, this management approach is not specific to COVID-19 patients [49]. Acutely ill or hospitalized COVID-19 patients, including those receiving critical care, were found to have high rates of venous thromboembolism (VTE). In these patients, the best thromboprophylaxis strategy is still uncertain [50]. Recently, the American Society of Hematology (ASH) guideline panel suggests using prophylactic-intensity over intermediate-intensity or therapeutic intensity anticoagulation for patients with COVID-19–related acute illness who do not have suspected or confirmed VTE [51]. Conte G et al. thought that recommendations for using pharmacological DVT prophylaxis are mostly based on the results of research with medical patients who did not have COVID-19. Furthermore, it is unclear whether antithrombotic prophylaxis in COVID-19 patients should be guided by risk assessment models (as was the traditional approach in nonsurgical hospitalized patients prior to the present epidemic), D-dimer values, or clinical judgment alone [52]. In a multicenter, randomized, controlled trial including patients hospitalized with confirmed COVID-19 and elevated D-dimer concentration, a 30-day course of therapeutic anticoagulation with rivaroxaban at 20 mg daily (and enoxaparin 1 mg/kg twice daily for clinically unstable patients) did not result in better clinical outcomes when compared with in-hospital prophylactic anticoagulation with heparin. Therapeutic anticoagulation for 30 days with rivaroxaban or enoxaparin led to a higher incidence of major or clinically relevant nonmajor bleeding than did inhospital prophylactic anticoagulation [53]. According to Al-Ani et al., the following anticoagulants hold great potential in reducing the frequency of microthrombi formation in COVID-19 patients [54].

1. Heparin: In their recent retrospective review, Tang et al. assessed the effect of heparin therapy on the mortality rate of COVID-19 patients. They reported that, overall, there was no significant difference in the mortality rates of COVID-19 patients who either received or did not receive heparin. However, the COVID-19 patients with abnormally high levels of D-dimer who received heparin exhibited significantly lower mortality rates than the COVID-19 patients with abnormally high levels of D-dimer but who did not receive heparin. The former result could be attributed to the fact that other clinical settings and treatment modalities received by the patients were not revealed, impacting the mortality of the COVID-19 patients [55, 56].
2. TPA: Although to date very few studies have assessed the effects of TPA administration on mortality rates of COVID-19 patients, their results have been positive. These studies have reported significant improvement in the ventilator parameters and oxygenation index of severely ill COVID-19 patients after TPA administration [55].
3. Direct oral anticoagulants (DOACs): This category of anticoagulants seems to be effective in COVID-19 patients based on the findings of Testa et al. who reported a marked increment in the plasma levels of DOAC in COVID-19 patients who received antiviral medications [57].

A recent study published by Spyropoulos et al. reported that the use of therapeutic-dose low molecular weight heparin (LMWH) for thromboprophylaxis in high-risk inpatients with COVID-19 decreased thromboembolism and mortality in their trial. High-dose anticoagulant therapy altered the course of illness in patients who were not in the intensive care unit but had a very high risk of thromboembolism and mortality (36.1% incidence in the standard dose group) [58].

## 5. Conclusion

In conclusion, it is clearly evident that levels of D-dimer are directly associated with the disease severity among COVID-19 patients. Novel coronavirus infections promote inflammatory and coagulation reactions, leading to an increase in thrombotic event rates. Currently, the evaluation of levels of D-dimer has not been adopted in the routine laboratory assessment of COVID-19 patients. Laboratory testing for D-dimer and proinflammatory cytokines could help to categorize the COVID-19 patients based on the severity of their illness. This could, in turn, be helpful in adequate and more efficient management of such individuals. We also recommend that future investigators should focus on conducting more comprehensive and multicentric prospective studies to elucidate further the association of levels of D-dimer with the levels of proinflammatory cytokines and with the thrombotic pathways, especially in COVID-19 individuals. Finally, we propose that physicians should consider using anticoagulant therapies to counter the upregulated thrombotic activity in COVID-19 patients.

## Figures and Tables

**Table 1:** A list of some retrospective studies showing the significance of the association of D-dimer levels with disease severity among the COVID-19 patients [13, 27, 35, 36, 37, 38, 39, 40, 41, 42, 43].

	Study population	Observations
Han et al. 2020	Control vs. COVID-19 patients	COVID-19 patients exhibited significantly higher levels of both D-dimer and fibrinogen
Zhou et al. 2020	COVID-19 survivors vs. nonsurvivors	Patients with D-dimer level >1 *μ*g/mL exhibited significantly (18 times) higher mortality than those with D-dimer level <1 *μ*g/mL (*p*=0.0033)
Cui et al. 2020	COVID-19 patients with VTE vs. those without VTE	A D-dimer level cut-off value of 1.5 *μ*g/mL could be used to predict VTE
Tang et al. 2020	COVID-19 survivors vs. nonsurvivors	About 28.36% and 85.7% of the COVID-19 patients with DIC exhibited fibrinogen level <1 g/L and D-dimer level >3 mg/dL, respectively
Qui et al. 2020	Mild COVID-19 vs. moderate COVID-19; all pediatric patients	Mild COVID-19 patients exhibited significantly lower levels of D-dimer compared to moderate COVID-19 patients
Liu et al. 2020	Mild COVID-19 vs. severe COVID-19	Mild COVID-19 patients exhibited significantly lower levels of D-dimer compared to severe COVID-19 patients
Zhang et al. 2020	Mild or moderate COVID-19 vs. severe COVID-19	Mild/moderate COVID-19 patients exhibited significantly lower levels of D-dimer compared to severe COVID-19 patients
Chen et al. 2020	Moderate COVID-19 vs. severe COVID-19	Moderate COVID-19 patients exhibited significantly lower levels of D-dimer compared to severe COVID-19 patients
Wu et al. 2020	COVID-19 patients with ARDS vs. those without ARDS	Risk of ARDS in COVID-19 patients directly correlated with the levels of D-dimer (*p* < 0.001)
Zhou et al. 2020	Patients with no aggravated COVID-19 vs. Patients with aggravated COVID-19	The pathological progression of COVID-19 was not impacted by the levels of D-dimer
Wu et al. 2020	COVID-19 survivors vs. nonsurvivors	Higher levels of D-dimer significantly correlated with higher mortality risk among COVID-19 patients who presented with ARDS (*p*=0.002)
Tang et al. 2020	COVID-19 survivors vs. nonsurvivors	(i) Multivariate analysis revealed the level of D-dimer to be independently associated with 28-day mortality
		(ii) Among the COVID-19 patients with levels of D-dimer >3.0 *μ*g/mL, those who did not receive heparin exhibited a significantly higher 28-day mortality rate compared to those who received heparin (*p*=0.017)
Yin et al. 2020	Individuals suffering from both severe pneumonia and COVID-19 vs. individuals suffering from severe pneumonia but not COVID-19	Among the COVID-19 patients with levels of D-dimer > 3.0 *μ*g/mL, those who did not receive heparin exhibited a significantly higher mortality rate compared to those who received heparin (*p*=0.017)
Zhang et al. 2020	COVID-19 survivors vs. nonsurvivors	(i) Among the patients with levels of D-dimer of more than 1 mg/L, about 81% of patients exhibited severe illness, and around 72% of patients reached the composite endpoints, that is, admission to ICU or death
		(ii) Higher levels of D-dimer correlated significantly with the severity of pneumonia among the COVID-19 patients and the risk of the patient reaching the composite endpoints, that is, admission to ICU or death (*p* < 0.001)

VTE, venous thromboembolism; DIC, disseminated intravascular coagulation; ARDS, acute respiratory distress syndrome.

## Data Availability

The data that support the findings of this review are available in the reference section.
